# Is Antiviral Treatment with Remdesivir at the Acute Phase of SARS-CoV-2 Infection Effective for Decreasing the Risk of Long-Lasting Post-COVID Symptoms?

**DOI:** 10.3390/v16060947

**Published:** 2024-06-12

**Authors:** César Fernández-de-las-Peñas, Anabel Franco-Moreno, María Ruiz-Ruigómez, Estibaliz Arrieta-Ortubay, Pablo Ryan-Murua, Carlos Lumbreras-Bermejo, Pablo del-Valle-Loarte, Oscar J. Pellicer-Valero, Rocco Giordano, Lars Arendt-Nielsen, Isabel Martín-Garrido, Juan Torres-Macho

**Affiliations:** 1 Department of Physical Therapy, Occupational Therapy, Physical Medicine and Rehabilitation, Universidad Rey Juan Carlos, 28922 Madrid, Spain; 2 Center for Neuroplasticity and Pain (CNAP), Sensory-Motor Interaction (SMI), Department of Health Science and Technology, Faculty of Medicine, Aalborg University, DK 9220 Aalborg, Denmark; rg@hst.aau.dk (R.G.); lan@hst.aau.dk (L.A.-N.); 3 Department of Internal Medicine, Hospital Universitario Infanta Leonor-Virgen de la Torre, 28031 Madrid, Spain; anaisabel.franco@salud.madrid.org (A.F.-M.); pabloryan@gmail.com (P.R.-M.); juan.torresm@salud.madrid.org (J.T.-M.); 4 Department of Internal Medicine, Hospital Universitario Doce de Octubre, 28041 Madrid, Spain; rryruiz@gmail.com (M.R.-R.); earrietao@gmail.com (E.A.-O.); carlos.lumbreras@salud.madrid.org (C.L.-B.); 5 CIBER de Enfermedades Infecciosas (CIBERINFEC), Instituto de Investigación Carlos III (ISCIIII), 28220 Madrid, Spain; 6 Department of Internal Medicine, Hospital Universitario Severo Ochoa, 28911 Madrid, Spain; pablo.valle@salud.madrid.org; 7 Image Processing Laboratory (IPL), Universitat de València, Parc Científic, Paterna, 46980 València, Spain; oscar.pellicer@uv.es; 8 Department of Oral and Maxillofacial Surgery, Aalborg University Hospital, DK 9100 Aalborg, Denmark; 9 Department of Gastroenterology & Hepatology, Mech-Sense, Clinical Institute, Aalborg University Hospital, DK 9100 Aalborg, Denmark; 10 Steno Diabetes Center North Denmark, Clinical Institute, Aalborg University Hospital, DK 9100 Aalborg, Denmark; 11 Department of Internal Medicine, Hospital Virgen del Rocío, 41012 Sevilla, Spain; isamartinga@gmail.com; 12 Department of Medicine, School of Medicine, Universidad Complutense de Madrid, 28040 Madrid, Spain

**Keywords:** antiviral, remdesivir, long-COVID, post-COVID-19

## Abstract

The aim of this study was to investigate the effects of administrating Remdesivir at the acute COVID-19 phase on developing post-COVID symptoms in previously hospitalized COVID-19 survivors by controlling factors such as age, sex, body mass index, and vaccination status. A case-control study was performed. Hospitalized COVID-19 survivors who had received intravenous Remdesivir during the acute phase (n = 216) were matched by age, sex, body mass index, and vaccination status with survivors who did not receive antiviral treatment (n = 216). Participants were asked to self-report the presence of any post-COVID symptom (defined as a symptom that started no later than three months after infection) and whether the symptom persisted at the time of study (mean: 18.4, SD: 0.8 months). Anxiety levels (HADS-A), depressive symptoms (HADS-D), sleep quality (PSQI), and severity/disability (FIC) were also compared. The multivariate analysis revealed that administration of Remdesivir at the acute COVID-19 phase was a protective factor for long-term COVID development (OR0.401, 95%CI 0.256–0.628) and specifically for the following post-COVID symptoms: fatigue (OR0.399, 95%CI 0.270–0.590), pain (OR0.368, 95% CI 0.248–0.548), dyspnea at rest (OR0.580, 95%CI 0.361–0.933), concentration loss (OR0.368, 95%CI 0.151–0.901), memory loss (OR0.399, 95%CI 0.270–0.590), hair loss (OR0.103, 95%CI 0.052–0.207), and skin rashes (OR0.037, 95%CI 0.005–0.278). This study supports the potential protective role of intravenous administration of Remdesivir during the COVID-19 acute phase for long-lasting post-COVID symptoms in previously hospitalized COVID-19 survivors.

## 1. Introduction

The rapid spread of the coronavirus disease 2019 (COVID-19) has provoked billions of infections and millions of deaths around the world. Due to the virulence of severe acute respiratory syndrome coronavirus 2 (SARS-CoV-2), several medications have been used for managing the acute phase of the infection [1]. Among these acute treatment medications, antivirals are commonly used in the acute COVID-19 phase since they inhibit SARS-CoV-2 replication and can prevent the severe form of the disease [2]. Several antivirals can be used during the acute COVID-19 phase, including Nirmatrelvir, Ritonavir, and Remdesivir, which are recommended by the COVID-19 Treatment Guidelines Panel of the World Health Organization (WHO) [3]. This recommendation is based on current evidence supporting that administration of Nirmatrelvir-Ritonavir or Remdesivir at the acute phase of SARS-CoV-2 infection is associated with a lower mortality rate, a shorter hospitalization stay, and COVID-19 severity [4]. The first one combines an oral SARS-CoV-2 protease inhibitor with antiviral activity against coronaviruses as an active substance (Nirmatrelvir) with a pharmacokinetic booster (Ritonavir) [5]. The proposed dosage to administer Nirmatrelvir-Ritonavir at the acute COVID-19 phase is estimated at 300 mg Nirmatrelvir/100 mg Ritonavir for five consecutive days [6]. The second one, Remdesivir, is an antiviral agent that acts as a nucleotide analogue and binds to viral RNA-dependent polymerase, which terminates RNA transcription prematurely, thus inhibiting viral replication of SARS-CoV-2 [7]. Remdesivir needs an intravenous administration, and the proposed dosage for administrating Remdesivir during an acute SARS-CoV-2 infection is 200 mg the first day and 100 mg per day for 5 consecutive days, depending on COVID-19 severity [8].

A second growing healthcare problem derived from the COVID-19 outbreak has been the development of long-lasting symptoms once the acute infection has passed. In fact, several attempts are being explored to prevent the development of long-lasting symptoms following COVID-19 [9]. The presence of long-lasting symptoms after the acute infection is called long-COVID [10] or post-COVID-19 condition [11]. Different meta-analyses reported that 25–30% of COVID-19 survivors still report symptoms one [12,13] and two [14,15] years after the infection. Therefore, the identification of preventive strategies to reduce the risk of developing long-term COVID is crucial [16]. A recent systematic review has identified that evidence supporting a potential protective effect of administrating antiviral medication, e.g., Remdesivir or Nirmatrelvir/Ritonavir, at the acute phase of SARS-CoV-2 infection is heterogeneous and conflicting [17]. This review identified three studies investigating the effect of Remdesivir on decreasing the risk of developing post-COVID in previously hospitalized COVID-19 survivors and found two studies reporting a protective effect [18,19] and one that did not [20]. These studies included samples receiving Remdesivir at the acute phase of the infection, ranging between 79 and 163 patients, but did not control for other factors associated with post-COVID symptoms such as sex, age, or previous co-morbidities [18,19,20]. Therefore, the aim of the current study was to investigate the effects of administering Remdesivir at the acute COVID-19 phase on the development of post-COVID symptoms in a cohort of previously hospitalized COVID-19 survivors by controlling confounder factors such as age, sex, body mass index, and vaccination status. We hypothesized that COVID-19 survivors who had received intravenous Remdesivir in the first days of the acute infection at the hospital would develop a smaller number of post-COVID symptoms than those who did not.

## 2. Methods

### 2.1. Study Design and Participants

We used a case-control design where a cohort of COVID-19 survivors who received intravenous Remdesivir at the acute SARS-CoV-2 infection was matched by age, sex, body mass index, and vaccination status with a cohort of hospitalized COVID-19 survivors who did not receive Remdesivir.

Two cohorts of subjects who were hospitalized due to the SARS-CoV-2 infection at four urban hospitals in Madrid (Spain) from September 2020 to March 2021 were included. The predominant SARS-CoV-2 variants that circulated at the time of hospitalization were the historical strain (20A.EU2) between September and December 2020 and Alpha variant (B.1.1.7) between January and March 2021. The diagnosis of SARS-CoV-2 infection should have been confirmed by a reverse transcription-polymerase chain reaction (RT-PCR) assay of the nasopharyngeal and oral swab samples, as well as clinical/radiological findings at hospital admission. The study was approved by the Institutional Ethics Committees of all institutions/hospitals (H12OCT23/418; HSO25112020; URJC0907202015920; HUIL/092-20; HCSC 20/495E). All participants provided informed consent prior to collecting data. The current study was conducted during the initial phase of COVID-19 vaccination programs.

The first cohort was formed by hospitalized COVID-19 survivors who received Remdesivir as antiviral treatment during the acute phase of infection at hospitalization, as follows: 200 mg the first day and 100 mg/day for 5 consecutive days or until discharge (if shorter than 5 days) for a maximum duration of 10 days [8].

The second cohort was formed by hospitalized COVID-19 survivors who did not receive Remdesivir as antiviral treatment during the acute phase of infection, matched by age, sex, body mass index, and vaccination status with the first cohort.

### 2.2. Data Collection

Demographic (age, gender, height, weight), clinical data (medical comorbidities, vaccination status), and hospitalization data (COVID-19 onset symptoms, intensive care unit (ICU) admission, days at the hospital, administration or not of Remdesivir) were collected from hospital medical records in both cohorts.

Participants who agreed to participate in the study were scheduled for a telephone interview by trained healthcare researchers. We used the definition of post-COVID-19 condition as proposed by Soriano et al. [11]: “The post-COVID-19 condition occurs in people with a history of probable or confirmed SARS-CoV-2 infection, usually three months from the onset of the infection, with symptoms that last for at least two months and cannot be explained by an alternative medical diagnosis”. Accordingly, participants were asked to self-report the presence of symptoms that appeared three consecutive months after hospitalization due to COVID-19 and whether the symptom persisted at the time of the study. Although participants were free to report any symptom that they suffered from, a predefined list of symptoms, including the most prevalent post-COVID symptoms such as fatigue, dyspnea, anosmia, ageusia, visual disturbances, pain symptoms, brain fog, hair loss, or concentration loss, was systematically used.

The Hospital Anxiety and Depression Scale (HADS) and the Pittsburgh Sleep Quality Index (PSQI) were used for evaluating anxiety/depressive symptomatology and sleep quality, respectively, since both can be properly assessed by telephone [21]. Both the anxiety (HADS-A, 7-items, 0–21 points) and depressive (HADS-D, 7-items, 0–21 points) scales of the HADS were used [22]. The HADS has shown good validity and reliability in individuals with long-term COVID [23]. A cut-off score of ≥8 points on each scale has shown good sensitivity and specificity for identifying anxiety or depressive symptoms [24]. The PSQI (0–21 points) assesses sleep quality during the previous month, where a cutoff of ≥8 points is considered indicative of poor sleep [25].

Finally, all participants fulfilled the Functional Impairment Checklist (FIC), an eight-item disease-specific questionnaire used for evaluating the functional consequences of SARS [26]. Four items assess symptoms including breathlessness at rest, breathlessness on exertion, general fatigue, and muscle weakness (FIC symptoms, 0–12 points), whereas the remaining four assess limitations in occupational daily living activities, leisure/social activities, basic daily living activities, and instrumental activities of daily living (FIC disability, 0–12 points) [26]. Each item is evaluated into four degrees of severity (0: no, 1: mild, 2: moderate, 3: severe). The FIC has shown good psychometric properties to be used in individuals with long-term COVID [27].

### 2.3. Statistical Analysis

Data was collected with STATA 16.1 and processed using Python’s library statsmodels 0.13.2; Scipy 1.7.3 was used for conducting the statistical tests and statsmodels 0.11.0 for performing *p*-value correction. Data are presented as the mean (standard deviation, SD) for quantitative data or the number of cases (percentage) for categorical data. We compared the differences in post-COVID symptoms among patients who had received Remdesivir and those who did not with Chi-squared or ANOVA tests as needed. The level of significance was set at a priori 0.05, with *p*-values being corrected by means of the Holm-Bonferroni correction. Further, adjusted odds ratios (OR) and confidence intervals (95% CI) were also calculated by using multivariate logistic regression models for the risk of developing post-COVID symptoms. A priori, the level of significance was set at 0.05. No Type I error correction was used for this analysis.

## 3. Results

From 300 previously hospitalized COVID-19 survivors (75 randomly selected from each hospital) who had received intravenous Remdesivir during the acute phase of infection and who were invited to participate, a cohort of 216 (mean age: 55.4, SD: 12.6 years, 43.5% women) were finally included. In addition, a cohort matched by sex, age, BMI, and vaccination status of 216 (age: 55.6, SD: 12.7 years, 43.5% women) previously hospitalized COVID-19 survivors who had not received Remdesivir or any other effective antiviral treatment during the acute phase of infection during hospitalization were included.

Table 1 shows clinical data for COVID-19 survivors who received intravenous Remdesivir and for those who did not. No differences were identified in clinical data between both groups, except that fever was more prevalent as a COVID-19-associated-onset symptom in patients who did not receive Remdesivir. The most prevalent associated-onset symptoms were fever, dyspnea, myalgia, and cough (Table 1).

Participants who had received intravenous Remdesivir during hospitalization were assessed at 18.3 (SD 0.7) months after hospital discharge, whereas patients who did not receive Remdesivir were assessed at 18.4 (SD 1.0) months after hospital discharge (*p* = 0.790). At the time of the study, 141 (65.3%) patients who received Remdesivir during hospitalization reported at least one post-COVID symptom, whereas 178 (82.4%) patients who did not receive administration of Remdesivir at hospitalization exhibited at least one post-COVID symptom (OR 0.401, 95%CI 0.256 to 0.628, *p* < 0.001). Thus, patients who had received Remdesivir during hospitalization reported a significant (*p* = 0.02) lower number of post-COVID symptoms (mean: 1.6, SD: 1.6) than those who did not receive administration of Remdesivir (mean: 2.1, SD: 1.4).

Table 2 shows post-COVID symptoms in COVID-19 survivors who had received Remdesivir and in those who did not. Fatigue, dyspnea at exertion, and pain symptoms were the most prevalent post-COVID symptomatology. Overall, patients who received administration of Remdesivir at hospitalization reported a lower prevalence rate of fatigue (*p* = 0.002), pain symptoms (*p* = 0.006), dyspnea at rest (*p* = 0.04), memory loss (*p* < 0.001), concentration loss (*p* = 0.03), hair loss (*p* < 0.001), skin rashes (*p* < 0.001), and diarrhea (*p* = 0.02) than those who did not receive intravenous administration of Remdesivir.

The multivariate analysis revealed, after adjusting for all variables, that administration of Remdesivir at the acute COVID-19 phase was a protective factor for the development of post-COVID fatigue (OR 0.399, 95%CI 0.270 to 0.590, *p* < 0.001), pain symptoms (OR 0.368, 95% CI 0.248 to 0.548, *p* < 0.001), dyspnea at rest (OR 0.580, 95%CI 0.361 to 0.933, *p* = 0.025), memory loss (OR 0.399, 95%CI 0.270 to 0.590, *p* < 0.001), concentration loss (OR 0.368, 95%CI 0.151 to 0.901, *p* = 0.03), hair loss (OR 0.103, 95%CI 0.052 to 0.207, *p* < 0.001), and skin rashes (OR 0.037, 95%CI 0.005 to 0.278, *p* = 0.001).

Additionally, patients who had received intravenous Remdesivir also showed lower severity of symptoms (FIC symptoms, *p* < 0.001), lower disability (FIC disability, *p* = 0.02), lower anxiety levels (HADS-A, *p* < 0.001), and lower depressive symptoms (HADS-D, *p* < 0.001) than those who did not receive Remdesivir (Table 2).

## 4. Discussion

This study found that COVID-19 survivors who received intravenous administration of Remdesivir at hospitalization exhibited a lower risk (OR 0.401, 95% CI 0.256–0.628) of developing long-lasting post-COVID symptoms than COVID-19 survivors who did not receive intravenous Remdesivir at the acute COVID-19 phase.

Current evidence on the protective role of antivirals administered during the acute phase of SARS-CoV-2 infection against long-term COVID is not clear [17]. Our results are in line with those previously observed by Boglione et al. [18] who also reported that administrating Remdesivir at the acute COVID-19 phase led to a reduction (OR 0.641, 95%CI 0.413–0.782) in the development of post-COVID symptoms six months after hospital discharge, but we disagree with Nevalainen et al. who did not show a significant effect on long-term COVID (RR 0.94, 95%CI 0.47–1.90) one year after hospitalization between COVID-19 survivors receiving Remdesivir and those who did not receive antiviral treatment [20]. Differences in study designs or follow-up periods could explain discrepancies between these studies. Thus, it is important to note that neither of these studies used a specific definition of long-term COVID or post-COVID-19 conditions [18,20]. Additionally, Nevalainen et al. concluded that, although they could not detect a statistically significant effect of Remdesivir on post-COVID symptoms, they obtained wide confidence intervals, including substantial benefit and harm [20]. The last possible explanation is the sample size. Nevalainen et al. included a sample of 181 participants [20], whereas Boglione et al. [18] included a sample of 462 participants, similar to our study. Accordingly, it is possible that the study by Nevalainen et al. was underpowered [20].

Another explanation could be related to the fact that the effects of antiviral medication may be symptom-specific since different post-COVID symptoms are mediated by various mechanisms. In fact, more than 100 symptoms affecting the cardiovascular, neurological, immune, respiratory, musculoskeletal, or gastrointestinal systems have been attributed to SARS-CoV-2 [28]. In such a scenario, some post-COVID symptoms may be influenced by the receipt of antivirals, whereas others may not. This hypothesis is supported by our results, where administration of Remdesivir was a protective factor, particularly for some of the most bothersome post-COVID symptoms such as fatigue, pain, dyspnea, memory loss, or concentration loss, but not for others including ageusia, anosmia, and gastrointestinal problems. Since antivirals aim to inhibit the replication of SARS-CoV-2 [7] a reduction in viral replication would lead to a reduced pro-inflammatory response, i.e., cytokine storm, and a lower host immune response. Accordingly, it is possible that administration of antivirals at the acute COVID-19 phase could be more effective for preventing those post-COVID symptoms more associated with this viral replication or a higher inflammatory response. This rationale could lead to the hypothesis that not all COVID-19 survivors will benefit from intravenous administration of Remdesivir and that potential subgroups could exist.

The use of antivirals during an acute SARS-CoV-2 infection has been associated with a lower mortality rate, hospitalization stay, and COVID-19 severity [4]. Accordingly, the protective effect of Remdesivir on the development of post-COVID symptoms could also be associated with a reduction in COVID-19 severity, a consequent reduction in hospital stay, a lower risk of non-invasive ventilation, and a lower ICU admission. In fact, Boglione et al. found that ICU admission and hospitalization time were those factors more associated with the development of post-COVID symptoms, and these authors attributed the effects of Remdesivir on long-term COVID to the fact that COVID-19 survivors treated with Remdesivir had a shorter hospitalization rate, a lower rate of hospital-related symptoms, and a lower ICU admission rate [18]. Our study did not support this hypothesis since both groups did not differ on ICU admission or hospitalization stay. Thus, no differences in hospitalization data between both cohorts in our study were seen, suggesting that these variables did not explain the protective effect of Remdesivir.

The results of the current study should be considered according to their strengths and limitations. The inclusion of a comparative group of COVID-19 survivors who did not receive Remdesivir compared to the group of COVID-19 survivors who received this drug and the long-term follow-up (one year and a half) after hospitalization can be considered strengths of the design; however, some limitations are also considered. First, we only included hospitalized cohorts; therefore, these results can only be applied to previously hospitalized patients. The use of hospitalized cohorts is based on the fact that Remdesivir is an antiviral drug needing intravenous administration, which is a potential disadvantage against other antivirals, such as Nirmatrelvir-Ritonavir, which can be administered orally. In fact, data from single studies on the effects of oral administration of Nirmatrelvir-Ritonavir is also controversial, as is intravenous administration of Remdesivir. For instance, Chuang et al. [29] found that treatment with Nirmatrelvir-Ritonavir during acute SARS-CoV-2 infection was not associated with decreased risk (OR 1.043, 95%CI 0.978–1.114) of post-COVID symptoms such as fatigue, pain, headache, dizziness, anxiety, depression, or sleep disorders in previously hospitalized COVID-19 survivors. On the contrary, Xie et al. [30] found that oral administration of Nirmatrelvir-Ritonavir reduced the risk of overall long-term COVID (RR 0.74, 95%CI 0.72–0.77) the first year after the acute infection in non-hospitalized COVID-19 survivors. It is important to consider that this study, in agreement with our study, also found that the effects of antivirals were more pronounced for some specific post-COVID symptoms such as fatigue, muscle pain, or dyspnea [30]. The STOP-PASC Randomized Clinical Trial has recently observed that oral administration of Nirmatrelvir-Ritonavir in COVID-19 survivors with post-COVID symptoms did not show a significant benefit to their symptoms [31]. Similarly, the effects of Nirmatrelvir-Ritonavir on post-COVID sequalae are also controversial since Wang et al. [32] found a decrease in the long-term risk of death and the development of post-acute cardiovascular and respiratory sequelae in a large sample of inpatient COVID-19 survivors, whereas Ioannou et al. [33] did not find this protective effect in a sample of non-hospitalized veterans. Second, the cross-sectional nature of our study did not permit us to identify the longitudinal evolution of post-COVID symptoms or the fluctuating nature of this condition. In addition, we used a case-control design, and a potential randomized clinical trial design could be used to confirm or refute the current result. Thus, post-COVID symptoms were self-reported. The use of specific patient-reported outcome measures (PROMs), e.g., the long-term COVID Symptom and Impact Tool [34], will be helpful to obtain more homogeneous post-COVID data. Third, although the dosage of Remdesivir is established by the FDA, we do not yet know if different dosages could have different protective effects on long-term COVID. Fourth, we did not collect biological variables, e.g., viral persistence, inflammatory mediators, and immune response, in addition to the post-COVID symptoms, which could help to further elucidate the potential mechanisms of Remdesivir and identify potential subgroups who will benefit from this antiviral drug.

## 5. Conclusions

This study found a lower risk of developing post-COVID symptoms (number and severity) in COVID-19 survivors who received intravenous Remdesivir at the acute COVID-19 phase during hospitalization than in those COVID-19 survivors who did not receive Remdesivir.

## Figures and Tables

**Table 1:** Clinical and Hospitalization Data according to oral administration or not of Remdesivir.

	No Remdesivir (n = 216)	Remdesivir (n = 216)	*p*-Value
Female n (%)	94 (43.5%)	94 (43.5%)	-
Age (years)	55.6 ± 12.7	55.4 ± 12.6	0.882
Weight (kg)	81.9 ± 15.2	80.8 ± 14.0	0.441
Height (cm)	167.4 ± 10.9	166.6 ± 9.2	0.409
Number of pre-existing co-morbidities	0.9 ± 0.9	0.8 ± 0.9	0.246
Obesity (pre-existing)	20 (9.25%)	28 (13.0%)	0.248
Hypertension (pre-existing)	64 (29.6%)	51 (23.6%)	0.225
Diabetes (pre-existing)	25 (11.6%)	9 (4.2%)	0.242
Asthma (pre-existing)	10 (4.6%)	11 (5.1%)	0.827
COPD (pre-existing)	7 (3.25%)	6 (2.8%)	0.781
Musculoskeletal Pain (pre-existing)	85 (39.35%)	64 (29.6%)	0.181
Cardiac diseases (pre-existing)	23 (10.65)	18 (8.3%)	0.435
Rheumatological diseases (pre-existing)	1 (0.45%)	0 (0.0%)	0.318
Other diseases (pre-existing)	38 (17.6%)	39 (18.05%)	0.909
Number of COVID-19 symptoms at hospital admission	2.4 ± 0.8	2.15 ± 1.0	0.09
Fever (COVID-19 onset) *	155 (71.75%)	102 (47.2%)	0.04 *
Dyspnea (COVID-19 onset)	73 (33.8%)	87 (40.3%)	0.268
Myalgias (COVID-19 onset)	89 (41.2%)	58 (26.85%)	0.412
Cough (COVID-19 onset)	60 (27.8%)	62 (28.7%)	0.856
Headache (COVID-19 onset)	46 (21.3%)	50 (23.15%)	0.683
Diarrhea (COVID-19 onset)	35 (16.2%)	14 (6.5%)	0.114
Anosmia (COVID-19 onset)	21 (9.7%)	39 (18.05%)	0.724
Ageusia (COVID-19 onset)	20 (9.25%)	18 (8.3%)	0.745
Throat pain (COVID-19 onset)	12 (5.5%)	18 (8.3%)	0.273
Vomiting (COVID-19 onset)	4 (1.85%)	0 (0.0%)	0.345
Dizziness (COVID-19 onset)	3 (1.4%)	9 (4.2%)	0.283
Days at hospital	12.9 ± 12.6	16.6 ± 12.4	0.365
ICU admission (yes)	28 (12.9%)	29 (13.4%)	0.816
Days at ICU	16.0 ± 14.5	13.0 ± 14.0	0.461
Vaccinated (yes)	68 (31.5%)	65 (30.1%)	0.805

* Significant differences between groups (*p* < 0.05).

**Table 2:** Post-COVID Symptoms and Psychological Symptoms according to oral administration or not of Remdesivir.

	No Remdesivir (n = 216)	Remdesivir (n = 216)	*p*-Value
Fatigue *	145 (67.1%)	97 (44.9%)	0.002 *
Dyspnea at exertion	116 (53.7%)	113 (52.3%)	0.843
Pain Symptoms *	114 (52.8%)	63 (29.2%)	0.006 *
Dyspnea at rest *	54 (25.0%)	35 (16.2%)	0.04 *
Memory Loss *	38 (17.6%)	0 (0.0%)	<0.001 *
Cognitive Blurring-Brain Fog	19 (8.8%)	21 (9.7%)	0.751
Concentration Loss *	18 (8.3%)	7 (3.2%)	0.03 *
Hair Loss *	69 (31.9%)	10 (4.6%)	<0.001 *
Palpitations-Tachycardia	14 (6.5%)	10 (4.6%)	0.414
Skin Rashes *	24 (11.1%)	1 (0.5%)	<0.001 *
Gastrointestinal Problems	15 (6.95%)	6 (2.8%)	0.495
Diarrhea *	9 (4.2%)	0 (0.0%)	0.02*
Voice Problems	4 (2%)	5 (2.35%)	0.739
Ageusia	7 (3.2%)	6 (2.8%)	0.761
Anosmia	7 (3.2%)	7 (3.2%)	0.999
Visual Disturbances	16 (7.4%)	9 (4.2%)	0.161
Throat Pain	7 (3.2%)	11 (5.1%)	0.342
FIC symptoms (0–12) *	3.2 ± 2.8	2.0 ± 2.4	<0.001 *
FIC disability (0–12) *	1.7 ± 2.4	1.0 ± 2.0	0.02 *
HADS-A (0–21)*	5.4 ± 5.2	1.3 ± 2.1	<0.001 *
Anxiety (HADS-A ≥ 8 points) *	70 (32.4%)	4 (1.85%)	<0.001 *
HADS-D (0–21) *	5.5 ± 4.9	1.45 ± 2.3	<0.001 *
Depression (HADS-D ≥ 8 points) *	80 (37.05%)	7 (3.2%)	<0.001 *
Sleep Quality (0–21)	6.5 ± 3.9	7.3 ± 4.0	0.421
Poor Sleep Quality (PSQI ≥ 8 points)	76 (35.29%)	77 (35.65%)	0.935

* Significant differences between groups (*p* < 0.05).

## Data Availability

All data are presented in the text.
